# Prenatal and Postnatal Nutrition Influence Pancreatic and Intestinal Carbohydrase Activities of Ruminants

**DOI:** 10.3390/ani11010171

**Published:** 2021-01-13

**Authors:** Ronald J. Trotta, Kendall C. Swanson

**Affiliations:** 1 Department of Animal and Food Sciences, University of Kentucky, Lexington, KY 40546, USA; ronald.trotta@uky.edu; 2 Department of Animal Sciences, North Dakota State University, Fargo, ND 58108, USA

**Keywords:** amylase, cattle, developmental programming, digestion, gestational nutrition, glucoamylase, isomaltase, lactase, maltase, sheep

## Abstract

**Simple Summary:**

Developmental programming is the concept that external influences that occur pre-conception, during gestation, or during early postnatal life can have long-term consequences for offspring growth, metabolism, and health. In ruminant livestock species, maternal diet is an important component influencing long-term programming of gastrointestinal function. Pancreatic and small intestinal digestive enzymes play an important role in postruminal digestion, primarily of carbohydrates and protein. This review will highlight current information regarding developmental programming of carbohydrases in response to dietary factors. Understanding how diet influences enzyme activity during early prenatal and postnatal life could lead to the development of dietary strategies to optimize offspring growth and development by increasing digestive efficiency of ruminant livestock species.

**Abstract:**

In ruminant livestock species, nutrition can play an important role in the long-term programming of gastrointestinal function. Pancreatic and small intestinal digestive enzymes are important for postruminal digestion of carbohydrates and protein. Carbohydrases have been shown to respond to changes in the level of feed intake and the dietary inclusion of specific nutrients, including arginine, butyrate, folic acid, fructose, and leucine. Understanding how diet influences enzyme development and activity during prenatal and postnatal life could lead to the development of dietary strategies to optimize offspring growth and development to increase digestive efficiency of ruminant livestock species. More research is needed to understand how changes in fetal or neonatal carbohydrase activities in response to nutrition influence long-term growth performance and efficiency in ruminant livestock species to optimize nutritional strategies.

## 1. Introduction

Maternal nutrition during gestation is a major determinant of fetal growth, development, and function [1] and nutrient restriction during gestation can have adverse effects on fetal visceral tissues [2,3]. Alterations in fetal visceral organ function during the prenatal phase can have negative effects on postnatal growth [4]. The ruminant gastrointestinal tract and liver constitute less than 10% of body weight (BW) but account for approximately 50% of total energy expenditure [5] and are major components defining maintenance requirements [6]. Changes in maternal and fetal visceral organ mass in response to nutrient restriction during gestation could potentially alter maintenance energy requirements of both the dam and fetus [7,8,9]. Programming of offspring maintenance energy requirements could be a useful strategy to optimize performance and efficiency in ruminants [10]. Interestingly, cows with low residual feed intake (most efficient) have decreased dry matter (DM) intake, greater ruminal passage rates, and decreased ruminal amylase and cellulase activities [11]. Therefore, increases in digestive enzyme activities, at least by the ruminal microbes, may not be necessary for improvements in whole animal efficiency. Digestive enzyme secretions have been estimated to consume nearly 25% of energy expenditure within the ruminant gastrointestinal tract [5,12]. While increased digestive enzyme activity could lead to greater substrate hydrolysis and nutrient digestibility, these improvements may not overcome the energy-demanding costs of increased protein synthesis, visceral organ mass, or enzyme secretion.

Optimization of digestive enzyme activity could potentially influence nutrient digestibility, whole-animal metabolism, and efficiency. In general, ruminants possess a high capacity to digest protein in the small intestine and absorb end-products (amino acids, di-, and tri-peptides) of small intestinal protein hydrolysis [13]. In contrast, research has suggested that the extent of small intestinal starch digestion is limited in beef cattle (55%) [14] and dairy cattle (60%) [15]. Host digestion of starch in the small intestine allows for absorption of glucose which provides more energy to the host than short-chain fatty acids produced from microbial fermentation of carbohydrates in the rumen [16]. Small intestinal starch digestion in cattle is therefore energetically more efficient than ruminal fermentation of starch [14,17,18]. Improvements in small intestinal starch digestibility in steers have been associated with increases in pancreatic and small intestinal carbohydrase activities [19,20,21].

The pancreas and small intestine have important roles in postruminal nutrient digestion and there is a limited amount of information on their function in response to nutritional adaptation [22]. Recent research on the effects of maternal and early postnatal nutrition on fetal or neonatal gastrointestinal function has improved our understanding of the regulation of digestive enzyme activity in ruminants. However, very few studies have evaluated the long-term potential for programming strategies to optimize digestive enzyme activity and nutrient digestion in ruminants. Understanding how diet can influence enzyme activity during prenatal and early postnatal life could lead to the development of dietary strategies to optimize offspring growth and development to increase digestive efficiency of ruminant livestock species. This review will summarize current research of developmental programming of pancreatic and small intestinal digestive enzymes involved in carbohydrate digestion in response to maternal diet or early postnatal diet in ruminant livestock species.

## 2. Carbohydrase Activity in Ruminants

Almost all of the carbohydrases involved in carbohydrate digestion are present in the intestinal mucosa and pancreas of ruminants but in less quantities than nonruminant animals [22,23,24,25,26,27]. Ruminants lack salivary α-amylase and intestinal sucrase but it is unclear how the absence of these enzymes influences carbohydrate digestion. It should be noted that cattle and buffalo possess nasolabial amylase [28] which might indirectly provide the functional role of salivary α-amylase in ruminants. Relative concentrations of pancreatic α-amylase and small intestinal maltase and isomaltase in cattle and sheep are much lower compared to nonruminants [23,25].

A simplistic, three-step model can be used to describe small intestinal starch digestion and absorption [29]: (1) hydrolysis of starch by pancreatic α-amylase into smaller oligosaccharides and limit-dextrins, (2) hydrolysis of small chain oligosaccharides into free glucose by brush border carbohydrases, and (3) glucose transport from the intestinal lumen into an absorptive enterocyte. Initiation of starch digestion in the small intestine begins with pancreatic α-amylase that is secreted into the intestinal lumen via the pancreatic duct. Pancreatic α-amylase is produced in pancreatic acinar cells and is secreted in its active form. In the lumen, pancreatic α-amylase hydrolyzes α-1,4-linked resident glucose molecules in starch and releases smaller oligosaccharides (maltose, maltotriose, and limit-dextrins) [30].

Four proteins in the ruminant small intestine possess carbohydrase activity: sucrase-isomaltase (SI), maltase-glucoamylase (MGAM), lactase (LCT), and trehalase (TREH). In pre-weaned ruminants, lactase hydrolyzes the β-1,4 glycosidic bond of lactose to produce glucose and galactose. Trehalase activity is present in the small intestinal mucosa but its nutritional significance in ruminants is unknown [22,31]. All four subunits of SI and MGAM contribute to intestinal starch hydrolysis in nonruminants and each subunit may hydrolyze more than one substrate [32]. In mice and humans, approximately 80% of the apparent maltase activity is derived from SI and the remaining 20% from MGAM [33,34]. Therefore, estimates of carbohydrase activity from the intestinal mucosa may have multiple protein subunits contributing to apparent carbohydrase activity. 

## 3. Influence of Prenatal Nutrition on Fetal Carbohydrase Activity

### 3.1. Maternal Diet Influences on Pancreatic Carbohydrase Activity

**Figure 1:**
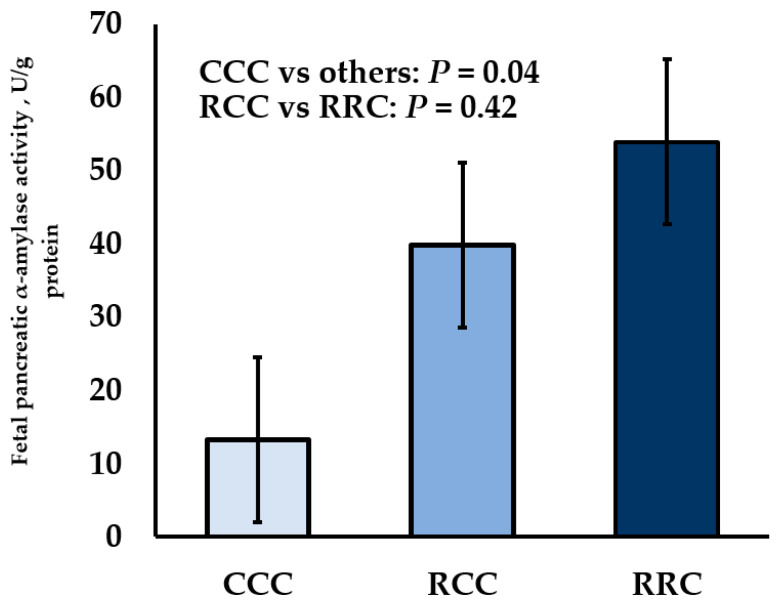
Influence of maternal dietary intake restriction (60% of NRC [39] recommendations) of cows during early-gestation (RCC; day 30 to day 85) or early- to mid-gestation (RRC; day 30 to day 140) on fetal pancreatic α-amylase activity per gram of protein (U/g protein) from samples collected during late-gestation (day 254). Abbreviations: CCC = cows fed to meet 100% of NRC [39] recommendations during gestation (day 30 to day 254); RCC = 60% maternal nutrient restriction of cows during early-gestation (day 30 to day 85), followed by realimentation to control diet during mid-to-late gestation (day 85 to day 254); RRC = 60% maternal nutrient restriction of cows during early-to-mid-gestation (day 30 to day 140), followed by realimentation to control diet during late gestation (day 140 to day 254). Adapted from Keomanivong et al. [36].

Various models of developmental programming have been studied, in which the dam is exposed to various stressors such as carrying multiple fetuses, maternal under- or overnutrition, changes in specific dietary components, maternal genotype, and heat stress [35]. Studies evaluating fetal programming of digestive enzymes have largely focused on changes in the level of DM intake of the dam through various periods of gestation. Maternal dietary restriction of beef cows during early- or early-to-mid-gestation and then realimentation to maintenance intake during late-gestation increased fetal pancreatic α-amylase per gram of protein by 3.0- and 4.1-fold, respectively (Figure 1); [36]. Furthermore, fetal pancreatic α-amylase per gram of protein was not influenced when cows were restricted during either early or early-to-mid-gestation. In sheep, maternal dietary restriction during mid-, late-, or mid-to-late-gestation did not influence fetal pancreatic α-amylase activity [37,38]. Therefore, there could be species-specific responses to maternal nutrient restriction when comparing cattle and sheep. Interestingly, intestinal glucose transport also has been shown to differ between cattle and sheep [13]. However, it should be noted that when Keomanivong et al. [36] found a programming response of fetal pancreatic α-amylase, maternal dietary restriction occurred during early or early- to mid-gestation. In general, organogenesis occurs during early-to-mid-gestation [3] and thus, functional alteration of fetal gastrointestinal organs may be more severe during early gestational nutrient restriction. Timing, length, and the degree of maternal nutrient restriction are important factors that might contribute to differences in maternal nutrient utilization maintenance energy requirements, and the severity of intrauterine growth restriction.

Few studies have evaluated the effects of maternal nutrition on postnatal offspring pancreatic α-amylase activity in ruminants. Maternal dietary restriction and rumen-protected arginine supplementation from mid-gestation to parturition did not influence pancreatic α-amylase activity of lamb offspring at d 54 of age [40]. Maternal protein supplementation (369 g/d) to beef cows from day 124 of gestation to parturition did not influence apparent total tract starch digestibility, small intestinal mass, or pancreatic α-amylase per gram of protein of offspring that were slaughtered after finishing [41]. 

Although most of the research pertaining to developmental programming of digestive enzymes in ruminants has focused on the level of maternal dietary intake, consumption of toxic endophyte-infected tall fescue seed by pregnant sheep has been shown to induce intrauterine growth restriction in ovine fetuses [42]. Feeding toxic endophyte-infected tall fescue seed to ewes during mid-to-late gestation decreased fetal pancreatic mass in sheep [34] but did not influence offspring pancreatic mass after wethers were finished [43]. Furthermore, maternal consumption of toxic endophyte-infected tall fescue seed during mid-gestation decreased proximal jejunal mass of offspring at slaughter [43]. Changes in tissue mass have been associated with changes in the total activity of digestive enzymes in ruminants [19]. It is unknown if offspring pancreatic or small intestinal digestive enzyme activities were influenced by maternal exposure to toxic endophyte-infected tall fescue seed. However, pancreatic α-amylase and small intestinal maltase, isomaltase, and glucoamylase activities of steers grazing toxic endophyte-infected tall fescue for 82 d did not differ from steers grazing non-toxic endophyte-infected tall fescue [44]. Maternal exposure to toxic endophyte-infected tall fescue seed during mid-to-late-gestation has been shown to decrease vasoactivity of uterine and umbilical arteries of pregnant sheep [45]. Changes in uteroplacental blood flow, fetal nutrient uptake, and nutrient transporter abundance or activity with maternal toxic fescue exposure might explain some of the underlying mechanisms behind fescue toxicosis models of intrauterine growth restriction. Further research is warranted to investigate the influence of toxic endophyte-infected tall fescue exposure on maternal and fetal pancreatic and small intestinal function in ruminants.

### 3.2. Maternal Diet Influences on Small Intestinal Carbohydrase Activity

Fetal brush border carbohydrases are imprinted early in development [46,47] and their activities have been shown to be variable in response to maternal nutrition. To our knowledge, no studies have evaluated the effects of early-gestational maternal nutrient restriction on fetal small intestinal carbohydrase activities in ruminants. However, Meyer et al. [3] demonstrated that maternal dietary restriction of beef cows during early-to-mid-gestation increased fetal jejunal proliferation at d 125 and intestinal vascularity at d 245. These authors suggested that early-gestational nutrient restriction of the dam programmed the fetal small intestine to improve intestinal function as related to growth and absorption, which may influence future offspring performance and efficiency [3].

Maternal dietary restriction (40% restriction) from mid-to-late-gestation (day 50 to day 130) did not influence fetal small intestinal maltase, isomaltase, or glucoamylase activities in sheep [38]. In another study, 40% maternal dietary restriction from mid-to-late-gestation (day 50 to day 130) decreased fetal maltase activity per gram of intestine in sheep [48]. Furthermore, fetal small intestinal isomaltase and glucoamylase activities were numerically lower with maternal dietary restriction. Discrepancies between these studies could be because of differences in the severity of intrauterine growth restriction. For example, when fetal small intestinal maltase concentration was found to be decreased with maternal dietary intake restriction, fetal BW and small intestinal mass were also decreased (13.8% and 21.2%, respectively) [7,49]. Whereas when Trotta et al. [38] found no differences in fetal small intestinal carbohydrase activities, fetal BW and small intestinal mass also did not differ compared to fetuses from ewes fed to meet NRC [50] recommendations. The fetal small intestine is relatively short in length compared to their maternal counterpart and therefore, differences in fetal intestinal sampling sites could be a potential explanation for variation in carbohydrase activities. 

Researchers have recently evaluated the effects of maternal folic acid supplementation throughout gestation on small intestinal mRNA expression of SI, MGAM, and LCT in lambs from differing litter sizes [51]. The study was designed with a 2 × 3 factorial arrangement of treatments where folic acid was supplemented throughout gestation at 0, 16, or 32 mg/kg of diet DM. After parturition, lambs from twin-bearing and triplet-bearing ewes were slaughtered for tissue collection. There was a folic acid supplementation × litter size interaction where maternal folic acid supplementation had a quadratic effect on duodenal LCT expression in twins, but no effects in triplets [51]. Additionally, duodenal SI expression was greater in triplet-born lambs compared to twin-born lambs. Duodenal or jejunal MGAM mRNA expression of lambs did not differ with maternal folic acid supplementation or litter size [51]. Inadequate fetal nutrient supply resulting from multiple pregnancies, as well as maternal folic acid supplementation, may modulate changes in small intestinal function through epigenetic modifications [51]. 

Lactase produced in the mammalian intestine is the most important carbohydrase during early postnatal life because lactose is the primary carbohydrate ingested [47]. However, the practical implications of programming for enhanced small intestinal lactase activity are not clear, as small intestinal lactase activity decreases with age in lambs, irrespective of diet [52]. Trotta et al. [38] found that maternal dietary restriction during mid-gestation followed by realimentation to 100% of NRC [50] recommendations during late-gestation increased fetal lactase activity per gram of intestine and per gram of protein. This may indicate that nutrient restriction during mid-gestation potentially could be used as a programming strategy to increase fetal lactase activity. However, the practical benefits of increases in fetal lactase activity are not clear, as greater than 90% of intestinal lactose supply is digested in the small intestine in neonatal ruminants [53]. Thus, improvements in fetal lactase activity may not result in improvements in growth performance post-partum. 

Few studies have evaluated the influence of maternal nutrition during gestation on small intestinal digestive enzyme activity of offspring postpartum. Maternal dietary restriction and rumen-protected arginine supplementation from mid-gestation to parturition did not influence jejunal maltase, isomaltase, or glucoamylase activities of lamb offspring at d 54 of age [40]. Likewise, Yunusova et al. [4] found that offspring from ewes that were intake restricted from mid-gestation to parturition did not differ in jejunal maltase activity at day 180 postpartum.

## 4. Influence of Postnatal Nutrition on Neonatal Carbohydrase Activity

### 4.1. Neonatal Diet Influences on Pancreatic Carbohydrase Activity

Nutrients such as carbohydrates, amino acids, and fatty acids could potentially modulate differences in digestive enzyme activity [22]. In nonruminants, pancreatic and small intestinal digestive enzymes typically respond proportionally to luminal substrate flows [54]. When energy intake is controlled, increasing intestinal starch flow decreases pancreatic α-amylase activity and increasing intestinal protein flow increases pancreatic α-amylase activity in functional ruminants [23,55,56]. Britt and Huber [57] evaluated pancreatic α-amylase activity in calves fed a carbohydrate-free diet (70% protein, 30% fat) and supplemented with different sources of carbohydrates (45% carbohydrate, 40% protein, 15% fat). Calves fed the carbohydrate-free diet had increased pancreatic α-amylase activity by ≥ 54.9% compared to calves fed diets formulated with lactose, galactose, or glucose [57]. Other forms of carbohydrates, such as fructose, did not influence pancreatic α-amylase activity of calves fed milk-replacer [58].

Amino acids have been thought to have an important role in the regulation of pancreatic exocrine function, potentially through modulations of mRNA translation, protein synthesis, post-translational processing, and/or neuroendocrine signaling. When leucine (1.435 g/L milk), phenylalanine (0.725 g/L milk), or a combination of leucine or phenylalanine (1.435 g leucine/L milk and 0.725 g phenylalanine/L milk) were supplemented to milk-fed calves, there was no influence on pancreatic α-amylase per gram of protein [59]. Similarly, increasing levels of leucine supplementation (0, 0.2, 0.6, 0.8 g/kg BW) to neonatal calves fed milk-replacer had no effect on pancreatic α-amylase activity [60]. In primary cell culture or tissue models using pancreatic acinar cells or slices, amino acids such as phenylalanine [61], leucine [62,63,64], and isoleucine [65] increased α-amylase release. Phenylalanine increases α-amylase activity in dairy calves and the initiation of mRNA translation through phosphorylation of ribosomal protein S6 kinase 1 (S6K1) and eukaryotic translation initiation factor 4E binding protein 1 (4EBP1) [62]. Leucine and isoleucine have been shown to increase α-amylase synthesis and phosphorylation of the mammlian target of rapamycin (mTOR) signaling pathway [62,65]. Proteomic analysis has suggested that leucine modulates increases in pancreatic α-amylase activity in dairy calves by increasing citrate synthase activity in the TCA cycle, ATPase activity and oxidative phosphorylation, and stimulating the general secretory signaling pathway in pancreatic acinar cells [66].

Ruminal fermentation leads to the production of short-chain fatty acids, which may also be important regulators of pancreatic exocrine function. Butyrate and isovalerate have been shown to increase α-amylase release from ovine pancreatic acinar cells in vitro [67,68]. Similarly, Swanson et al. [69] found that greater pancreatic α-amylase abundance and activity from feeding a high starch/high energy diet to lambs was also associated with greater ruminal proportions of butyrate and isovalerate. Increasing levels of isovalerate supplementation (0, 3, 6, 9 g/d) linearly increased luminal α-amylase and lactase concentrations in pre-weaned dairy calves fed milk [70]. After weaning, supplementation of isovalerate also linearly increased luminal α-amylase concentration in dairy calves fed a 60% forage:40% concentrate diet [67]. Replacing flavomycin with sodium butyrate in milk-replacer (3 g/kg of DM) did not influence pancreatic α-amylase activity of steers at slaughter after a finishing period [71]. Ruminal fermentation of diets containing moderate to high concentrations of starch may moderate changes in short-chain fatty acid profiles to favor butyrate and isovalerate. Consequently, these short-chain fatty acids may aid in generating a stimulus for increased pancreatic α-amylase to prepare for enhanced postruminal starch flows. However, it should be noted that short-chain fatty acids may modulate pancreatic α-amylase secretion in vivo through acidification of the duodenal digesta [72]. It is unclear if butyrate or isovalerate supplementation to pre-weaned ruminants could influence pancreatic α-amylase activity post-weaning. Long-term studies on the effects of early postnatal nutrition on postruminal carbohydrase activities are needed to understand if changes in carbohydrase activities persist in later life. 

### 4.2. Neonatal Diet Influences on Small Intestinal Carbohydrase Activity

Individual nutrients such as carbohydrates, amino acids, and fatty acids have been evaluated for their effects on postruminal digestive enzyme activites as components of early postnatal diets (Table 1). Dietary carbohydrate composition could potentially influence small intestinal digestive enzyme activity in neonatal ruminants. In neonatal calves fed milk-replacer, 18% replacement of lactose with maltodextrin, maltodextrin with a high degree of α-1,6 branching, and maltose decreased jejunal maltase activity per gram of protein [53]. Furthermore, jejunal isomaltase activity per gram of protein decreased in response to greater amounts of maltodextrin or maltodextrin with a high degree of α-1,6 branching [53]. Dietary fructose supplementation (2.2 g/kg of BW) to neonatal calves fed milk-replacer increased glucoamylase activity per gram of intestine by 30% and *MGAM* mRNA expression by 6.8-fold after 28 d of feeding [58]. Furthermore, dietary fructose supplementation did not influence small intestinal maltase or isomaltase activities. In mice and humans, approximately 80% of the apparent maltase activity is derived from SI and the remaining 20% from MGAM [33,34]. Therefore, differential regulation of *MGAM* (increase) and *SI* (decrease) mRNA expression with dietary fructose supplementation may explain why there was no change in maltase activity yet an increase in glucoamylase activity [58].

Recent studies have evaluated the effects of dietary short-chain fatty acid supplementation on small intestinal development and function in neonatal calves. Increasing levels of isovalerate supplementation (0, 3, 6, 9 g/d) linearly increased lactase concentrations in pre-weaned dairy calves fed milk [70]. Dietary sodium butyrate supplementation (0.3% in milk-replacer) has been shown to increase jejunal lactase activity in dairy calves [75]. Koch et al. [73] evaluated the effects of the level of milk-replacer intake [restricted (6 L/d) or ad libitum (12 L/d)] and butyrate supplementation (0 or 0.24% of milk-replacer powder) on small intestinal carbohydrase mRNA expression and activity in dairy calves. Milk-replacer intake and butyrate supplementation did not influence mRNA expression of small intestinal *LCT*, *MGAM*, or *SI* in calves [73]. Their findings demonstrate that the level of DM or energy intake does not influence small intestinal carbohydrase mRNA expression in calves. Additionally, these authors found that the level of milk-replacer intake or butyrate supplementation at 0.24% of diet DM did not influence small intestinal maltase or lactase activities [73]. 

In functioning ruminants, postruminal protein flow has been directly (from abomasal/duodenal infusion) and indirectly (from dietary components) shown to stimulate postruminal carbohydrase activities [20,21,31] and small intestinal starch digestibility [76,77,78]. In preruminant dairy calves, partial replacement of skim milk powder with soy protein isolate, partially hydrolyzed soy protein isolate, or potato protein isolate did not influence small intestinal maltase per gram of protein [79]. Similarly, replacement of casein with soy protein isolate in milk diets fed to goat kids did not influence jejunal maltase per gram of protein [80]. However, replacement of casein with soy protein isolate (80% of casein N) supplemented with amino acids (20% of casein N) resulted in increased jejunal maltase specific activity of goat kids compared to the casein and soy protein isolate-based milk diets [80]. Others have evaluated the effects of heat-treated canola meal (34% of DM) and glycerol inclusion (5% of DM) of calf starter on small intestinal digestive enzyme activity. Heat-treated canola meal inclusion or glycerol inclusion in calf starter did not affect small intestinal lactase or maltase activities of ruminating calves [81].

Information about dietary amino acid supplementation to neonatal ruminants on small intestinal carbohydrases is scarce. Cao et al. [82] found that dietary leucine or phenylalanine had no influence on lactase activity in small intestinal digesta from calves fed milk and starter. However, increasing levels (0, 0.4, 0.6, or 0.8 g/kg of BW) of leucine supplementation to calves fed milk-replacer showed a quadratic effect on intestinal lactase with the 0.4 g leucine/kg of BW treatment being lower than all other treatments [60]. Additionally, increasing levels of supplemental leucine linearly decreased maltase and isomaltase activities. Leucine supplementation at 2.9% of DM to neonatal lambs for 42 d in milk-replacer decreased small intestinal maltase and isomaltase activities at slaughter after an 82-d finishing period [74]. Although these studies [60,74] were conducted in different species (calves vs. lambs) and dietary N concentrations were not balanced across treatments, there is an indication that long-term developmental programming of small intestinal digestive enzymes via neonatal nutrition could be possible in ruminants. Studies evaluating long-term effects of postnatal nutrition are needed to understand if changes in neonatal carbohydrase activities remain during later life.

To our knowledge, Dollar and Porter [83] were the first to report the absence of sucrase activity in young calves. Furthermore, no measurable sucrase activity was detected in mucosa or small intestinal digesta contents from lambs [23]. Later reports by Huber et al. [84] and Siddons [25] corroborated the findings that sucrase activity is absent from the digestive tract of the young calf. Shirazi-Beechey et al. [85] attempted to measure sucrase activity in isolated brush-border membrane vesicles from lamb intestine and received the same results as others. In humans, dietary sucrose or fructose increases sucrase activity in the small intestine [86] and dietary fructose supplementation can induce sucrase activity in patients with congenital sucrase-isomaltase deficiency [87]. However, dietary fructose supplementation to neonatal calves does not induce sucrase activity in the small intestine [58]. We are not aware of practical methods to induce sucrase activity in pre-weaning ruminants from either maternal or early postnatal influences.

## 5. Conclusions

Maternal nutrition during gestation and early postnatal nutrition can influence postruminal carbohydrase activity in cattle and sheep. While most of the current research has focused on changes in the level of DM intake, several studies have shown that individual nutrients such as amino acids, carbohydrates, and short-chain fatty acids can influence carbohydrase activity in young ruminants. Long-term responses of the gastrointestinal tract to prenatal and postnatal nutrition should be incorporated into developmental programming strategies to optimize the performance and efficiency outcomes of offspring. More research is needed to evaluate the effects of early-gestational maternal nutrition on maternal and offspring digestive enzyme activity in ruminants. Additional work is needed to understand if changes in fetal/neonatal digestive enzyme activities influence long-term programming of digestive enzymes, postruminal digestibility, and animal efficiency.

## Figures and Tables

**Table 1:** Selected studies evaluating the effects of dietary supplementation to neonatal calves or lambs on small intestinal carbohydrase activities ^1^.

Item	Study			
	Trotta et al. [58]	Reiners et al. [60]	Koch et al. [73]	Reiners et al. [74]
Species	Calf	Calf	Calf	Lamb
Age at slaughter	42 d	39 d	80 d	163 d
Feeding length	28 d	28 d	52 d	42 d
Diet	Milk-replacer	Milk-replacer	Milk-replacer	Milk-replacer
Supplement	Fructose	Leucine	Butyrate	Leucine
Amount	2.2 g/kg BW	0.2, 0.4, 0.8 g/kg BW	0.24% of DM	2.9% of DM
Maltase	→	linear ↓	→	↓
Isomaltase	→	linear ↓	-	↓
Glucoamylase	↑	→	-	→
Lactase	→	quadratic ^2^	→	→

Abbreviations: BW = body weight; DM = dry matter. ^1^ Response: ↑ = increase; ↓ = decrease; → = no change. ^2^ Supplementation of 0.4 g leucine/kg BW decreased small intestinal lactase activity compared to all other treatments.

## Data Availability

Not applicable.
